# Individual Differences in the Frequency-Following Response: Relation to Pitch Perception

**DOI:** 10.1371/journal.pone.0152374

**Published:** 2016-03-25

**Authors:** Emily B. J. Coffey, Emilia M. G. Colagrosso, Alexandre Lehmann, Marc Schönwiesner, Robert J. Zatorre

**Affiliations:** 1 Montreal Neurological Institute, McGill University, Montreal, Canada; 2 Laboratory for Brain, Music and Sound Research (BRAMS), Montreal, Canada; 3 Centre for Research on Brain, Language and Music (CRBLM), Montreal, Canada; 4 Department of Psychology, University of Montreal, Montreal, Canada; 5 Department of Otolaryngology Head & Neck Surgery, McGill University, Montreal, Canada; Birkbeck College, UNITED KINGDOM

## Abstract

The scalp-recorded frequency-following response (FFR) is a measure of the auditory nervous system’s representation of periodic sound, and may serve as a marker of training-related enhancements, behavioural deficits, and clinical conditions. However, FFRs of healthy normal subjects show considerable variability that remains unexplained. We investigated whether the FFR representation of the frequency content of a complex tone is related to the perception of the pitch of the fundamental frequency. The strength of the fundamental frequency in the FFR of 39 people with normal hearing was assessed when they listened to complex tones that either included or lacked energy at the fundamental frequency. We found that the strength of the fundamental representation of the missing fundamental tone complex correlated significantly with people's general tendency to perceive the pitch of the tone as either matching the frequency of the spectral components that were present, or that of the missing fundamental. Although at a group level the fundamental representation in the FFR did not appear to be affected by the presence or absence of energy at the same frequency in the stimulus, the two conditions were statistically distinguishable for some subjects individually, indicating that the neural representation is not linearly dependent on the stimulus content. In a second experiment using a within-subjects paradigm, we showed that subjects can learn to reversibly select between either fundamental or spectral perception, and that this is accompanied both by changes to the fundamental representation in the FFR and to cortical-based gamma activity. These results suggest that both fundamental and spectral representations coexist, and are available for later auditory processing stages, the requirements of which may also influence their relative strength and thus modulate FFR variability. The data also highlight voluntary mode perception as a new paradigm with which to study top-down vs bottom-up mechanisms that support the emerging view of the FFR as the outcome of integrated processing in the entire auditory system.

## Introduction

The scalp-recorded frequency-following response (FFR) to complex sounds [1] may present a paradox: whereas it is thought to capture how the auditory system represents basic features of sound with high fidelity [2–4] and reliability [5,6], several of its features vary considerably between listeners, even amongst a homogenous sample of young, healthy adults [7]. This inconsistency is surprising, because subtle variations in the frequency content, temporal precision, and inter-trial consistency of FFRs have been linked to enhanced processing in expert groups like musicians (e.g. [8–10]), and are sufficiently sensitive to be useful as biomarkers of deficient sound encoding in auditory processing and learning disorders [3,7,11–14].

This individual variation raises questions about what the FFR *means*; that is, which auditory information and cognitive processes are represented in the FFR, and by extension, which of those processes are being modulated by behavioural tasks and contribute to the differences observed in health and pathology. We address these questions by exploring how variability in the FFR is related to the perceptual phenomenon known as the 'missing fundamental' [15–17], to explore their mutual connections to pitch perception: because pitch is a perceptual phenomenon rather than a direct reflection of the physics of sound in the external environment, its neural representation should reflect the subjects' experience rather than the sound itself.

The FFR reflects the brain's response to sustained periodic sound. It is thought to originate in a combination of subcortical auditory nuclei (supporting work from animal studies is summarized in [2]), although recent evidence shows that there is also a cortical source [18]. The FFR closely resembles a low-pass filtered version of the eliciting sound, although the FFR's frequency composition does not always match that of the sound to which it responds: energy is present at the fundamental frequency (f0) when none is present in the physical stimulus [19]. The amplitude of f0 and harmonic representations covary with behaviour independently and have been hypothesized to represent an early distinction between two streams of auditory information [20]. Our goal is to determine if inter-individual variability in f0 strength is related to inter-indivual variability in perception.

Relatively straightforward relationships between f0 amplitude and expert behaviour have been reported. For example, stronger f0 representations and f0-tracking are positively related to measures of musicianship, and are thought to index training-induced enhancements [8,21,22] (although it should be noted that between-group f0 peak amplitude differences have not been found in every study, e.g. [23,24]). Conversely, f0 amplitude is negatively related to autism [25]. Furthermore, the extent of FFR suppression elicited by a competing task was related to behavioural performance in a unidirectional fashion [26]. However, the f0 also frequently demonstrates more complex relationships to behaviour. When people learned to segment a sequence of sounds based on its statistical characteristics, some showed an increase in f0 strength while others showed a decrease; those with an increase demonstrated better behavioural performance [27]. When people attended to one of two streams of auditory information, they again showed a range of neural responses from f0 enhancement to suppression; but rather than the direction, it was the amplitude of the modulation that best explained performance on a related behavioural task, such that greater neural modulation was related to *poorer* scores [28]. In a study using fMRI as well as EEG, neural responses to repeated sound ranged from repetition suppression to repetition enhancement, and BOLD fMRI suppression in the inferior colliculus was related to lower-amplitude but higher-fidelity FFRs (as measured by stimulus-to-response correlation); this was in turn related to more successful learning of non-native speech patterns [29]. The observation that increases in FFR f0 strength can be linked to both better and worse behavioural results further illustrate our incomplete understanding of the f0's role in sound processing.

Pitch encoding varies between people. When a neurologically healthy population is presented with complex harmonic sounds that lack energy at their fundamental frequencies (i.e. missing fundamental tone; MF), some people tend to report the higher frequency spectra that are physically present ('spectral' or 'analytic' listeners; SP listeners), whereas others perceive the missing fundamental ('fundamental' or 'synthetic' listeners; f0 listeners) [15–17,30,31]. This perceptual bias has neural correlates that support a role for the auditory cortex in missing fundamental perception [30]: patients with right temporal-lobe excisions encroaching onto Heschl's gyrus have difficulty perceiving the missing fundamental [32], asymmetry in grey matter volume in lateral Heschl's gyrus is related to f0 vs SP bias [33], and electrophysiological responses of the primary auditory cortex during the first 100 ms after sound onset differ according to perceptual bias [33,34]. Perceptual bias also appears to have a more flexible aspect. Some studies have reported a fundamental bias in musicians, which may have been caused by musical practice shifting the perceptual focus from SP to f0 perception [35], though this may depend on the nature of the musical experience including the type of instrument [34]. It has recently been shown that the number, order, and frequencies of the harmonics can influence f0/SP perception [31,33], with idiosyncratic yet relatively stable differences between individuals for specific stimuli [31]. Perception can also be influenced by the listening context, such as the harmonic relationship of a tone to preceding tones [31,36]. Furthermore, f0 perception in spectrally-biased listeners can be induced through repeated exposure and training on a specific MF stimulus [37,38]. This effect was observed only in the spectral-to-fundamental direction [38,39], and was accompanied by increased low gamma band (24–48 Hz) activity, bilaterally [38]. Gamma band synchronization is implicated in a wide range of cognitive tasks, and is thought to be a fundamental aspect of inter-region communication and function within the brain (for a recent review, see [40]). In this context, it may represent the neural correlate of the spectral information being combined into a coherent Gestalt-like percept of the missing fundamental [38].

The fundamental frequency of a sound is associated with, but not identical to, the perceptual experience of pitch [41–43]. The amplitude of the FFR's f0 appears to reflect how well individuals represent this important sound property and their ability to perceive pitch [44–48]. However, it has been demonstrated using frequency-shifted complex tones and dichotic presentation of alternating harmonics that the FFR's f0 represents information relevant to pitch processing but is not a direct representation of pitch [49]. The pitch computation itself may take place in the auditory cortex, where regions that represent pitch in an invariant way have been observed [50–52]. A rostral brainstem computation, observable using a horizontal rather than a vertical electrode montage, has also been proposed [53]. Peripheral explanations such as cochlear non-linearities have been ruled out because the missing fundamental percept is heard even when the frequency region is masked with noise [54].

Because both the FFR f0 strength and perceptual mode bias vary considerably between subjects [7,31] and pertain to the representation of pitch, we wanted to test the hypothesis that there is a relationship between the two. Although work on the locus of the pitch computation is incomplete [55], pitch encoding offers a way of studying the function of densely interconnected higher and lower level components of the human auditory system as a whole [56]; we therefore also wished to compare the FFR f0 with known cortical functional correlates of MF perception (i.e. gamma range activity). With the aim of gaining insight into both the brain's pitch encoding and extracting mechanisms and the meaning the FFR f0 amplitude variability with respect to perception and task demands, we conducted two experiments. In the first experiment, we presented complex tones with and without f0 energy and evaluated subjects' perceptual bias in order to test the hypotheses that i) f0 strength in the MF condition is positively correlated with the subject's propensity to hear in the fundamental mode; and that ii) the presence of f0 energy in the stimulus affects the FFR's f0 strength. We also tested several secondary questions: whether the f0 strength was related to musicianship, whether the mastoid-to-mastoid electrode montage can be used to observe pitch processing as suggested in early work [53], and how comparing the two electrode montages in common use might contribute to our understanding of inter-individual variability.

In the second experiment, we addressed whether differences in f0 strength might be caused by top-down influences of directed attention in real time, using a sensitive within-subjects design. Using short MF melodies, we tested the hypotheses that iii) subjects can learn to reversibly switch between perceptual modes, thus voluntarily selecting between low-level sound representations; that iv) f0 representation is related to accuracy scores on a behavioural measure of perceptual mode switching and differs when subjects are perceiving one or the other mode, indicating behaviourally-relevant top-down modulation of these representations; and that v) cortical gamma band activity is affected reversibly by condition in parallel. We evaluate both group and intraindividual differences between conditions for both experiments (hypotheses ii) and iv)) due to the complexity of relationships to behaviour that have been reported in literature. Because relationships between experience and both MF perception bias and FFR f0 amplitude have been reported (e.g. [8,34]), we further test how measures of musical experience are related to each of the physiological and behavioural measures.

## Experiment 1

### Materials and Methods

#### Participants

We recruited 39 healthy adults (mean age = 28.8 years, SD = 9.1, maximum age = 58); 22 were female, and 33 were right-handed. Written informed consent was obtained and experimental procedures were approved by the Montreal Neurological Institute Research Ethics Board and the Research Ethics Committee of the Faculty for Arts and Sciences of the University of Montreal.

All subjects reported having normal hearing and no neurological conditions. Data about musical and linguistic history were collected via an online survey (Montreal Music History Questionnaire; MMHQ; [57]. The mean total instrumental and vocal practice and lesson hours was 4365 (SD = 7491), and subjects with musical experience started training between 3 to 18 years (mean = 8.1, SD = 3.9). Subjects reported a variety of current main musical activities (Keyboard: 3, Voice: 5, Percussion: 2, Strings: 7, Woodwinds: 1, Electronic/computer-based production: 2, Other: 1, No current activity: 18).

Three subjects were native tonal language speakers. The first 30 subjects recruited demonstrated a fundamental bias, we therefore used the behavioural task to screen an additional 30 subjects and selected from among them 9 additional subjects who were not 'pure fundamental' listeners. We thereby obtained a range of behavioural scores for correlational analysis.

#### Study design

Subjects completed the MMHQ before the experiment. Prior to the EEG session, they performed the pitch mode perception task, which was a computerized listening task designed to measure a general propensity to hear pitch either in the fundamental or spectral mode (~12 mins). Subjects were then prepared for EEG and instructed to relax, listen to the stimuli, and remain still. Prior to recording the FFR to missing fundamental and fundamental-present sounds (~20mins), we recorded auditory brainstem responses to 4000 clicks (~3 mins) to screen out retrocochlear abnormalities in the auditory system (the presence and approximately similar latency of waveforms I, III and V was observed for all subjects).

#### Pitch mode perception task

**Fig 1:**
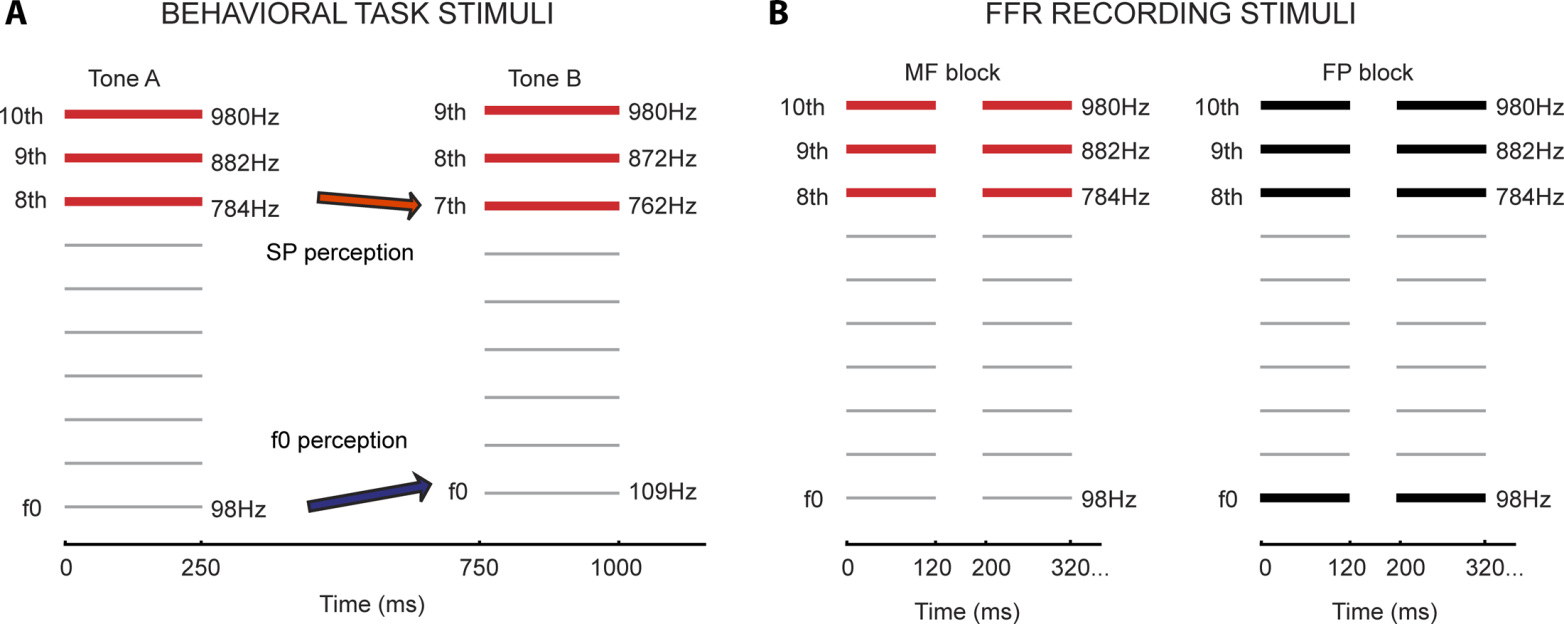
Experiment 1 experimental paradigm. (A) Behavioural testing of each listener’s perceptual bias. A sample tone pair is shown schematically; two complex missing fundamental tones comprised of the 8th-10th and 7th-9th harmonics were played sequentially after a short silent pause. This was repeated once, then subjects were asked to record whether they perceived the second tone to be higher or lower than the first. Tones were constructed such that spectral and fundamental perceptions lead to opposite responses, and a measure of overall perceptual bias was calculated from responses on 20 tone pairs. (B) Stimuli used in ABR testing; MF and FP stimuli differed only in the absence (MF: missing fundamental) or presence (FP: fundamental present) of energy at the fundamental frequency (f0).

To obtain a global index of subjects' perceptual preference, we adapted a commonly used task in which subjects listen to two consecutive harmonic complex tones that lack energy at the fundamental frequency and judge whether the second was higher or lower in pitch as compared to the first [31,33,35]. The tones are devised such that spectral and fundamental mode perceptions yield opposite answers (Fig 1(A)); to accomplish this, one of the tones contains a series of partials that is one harmonic number greater than the other (e.g. 7^th^ to 9^th^ vs. 8^th^ to 10^th^), and the frequency of the highest partial is kept constant to minimize the perception of edge pitch. The fundamental frequencies of the 20 tones were: 90, 98, 107, 111, 117, 132, 137, 141, 147, 153, 155, 159, 161, 163, 168, 170, 172, 176, 178, and 180 Hz. Subjects received the written instruction, 'You will hear two tones, which will then be repeated. Please indicate if the second tone appears to be lower (“L”) or higher (“H”) than the first. If you aren’t sure, please guess.' Subjects were also told that there is no right or wrong answer and that they should go with their stronger impression.

Complex relationships exist between perceptual mode preference and stimulus properties [58]: raising the lowest harmonic tends to produce more SP responses, whereas increasing the number of harmonics produces more f0 responses. Changing the average spectral frequency appears to influence the results, but in a non-linear fashion [31,33], and the harmonic relationship between the presented tones can affect how it is perceived [34]. Fundamental frequency, harmonic number and the number of harmonics are interrelated such that if the top frequency is kept steady it is not possible to vary the other parameters independently. We used a subset of stimuli similar to those used previously to reduce the dimensions (i.e. number of partials, harmonic number, and frequencies), reduce task length, and maximize similarity to the stimulus used to evoke FFRs. All stimuli had three partials; this is the midpoint of those characterized in Schneider et al. and has also been used in subsequent work [31]. We used the 7^th^-9^th^ and 8^th^-10^th^ harmonics of stimuli with fundamental frequencies in the range that produce strong FFRs (f0: 80-500Hz; [59], and top harmonic frequencies ranging from 900-1800Hz (a sample tone pair is illustrated in Fig 1(A)).

The task comprised 20 stimulus pair trials presented in counterbalanced order. The set of trials was presented twice in pseudo-randomized sequences. Each trial consisted of a 250 ms tone, a 500 ms gap, and a second 250 ms tone; this was then repeated once after a 1000 ms delay to allow subjects to confirm their initial judgement (see Fig 1(A)). Stimuli were constructed by summing equal amplitude cosine-phase tones (fade-in/fade-out of 10ms raised cosine ramp), using custom Matlab scripts (The Mathworks Inc., MA, USA) from which sound files were produced (WAV, 44.1kHz sampling rate). Subjects listened to stimuli through headphones (Sony MDR-V500 headphones), and recorded their responses ('H' or 'L'). Before the main experiment, subjects completed 10 practice trials in which both the f0 and SP percepts occurred in the same direction (i.e. different f0 frequencies, but the same harmonic numbers). This served to familiarize subjects with the procedure and to ensure they had normal pitch processing abilities (100% accuracy) in the frequency range of interest; all subjects passed.

The overall perceptual bias of each listener can be quantified as the proportion of f0 and SP responses on a scale from -1 to 1; (SP -f0) / (SP + f0), where f0 refers to the number of fundamental responses, and SP refers to the number of spectral responses. The valence of this measure has been used inconsistently in literature; here, negative values represent more fundamental answers [30,31,34] rather than the reverse [58]. We evaluated the relationship between perceptual bias and neural correlates and with musical experience using non-parametric statistics due to the non-normal distributions.

#### Data acquisition

EEG data were recorded in a magnetically shielded audiometric booth from monopolar active Ag/AcCl electrodes placed at Cz (10–20 International System), Fz (approximate; hairline), C7 (7^th^ vertical vertebra), and both mastoids, using an averaged reference (*BioSemi*, www.biosemi.com*)*. Two ground electrodes were placed above the right eyebrow. Because active electrodes reduce nuisance voltages caused by interference currents by performing impedance transformation on the electrode, we confirmed that direct-current offset was close to zero during electrode placement instead of measuring impedance. Electrode signals were amplified with a BioSemi ActiveTwo amplifier, recorded using open filters with a sampling frequency of 16384Hz, and stored for offline analysis using BioSemi ActiView software.

The main analyses were conducted using electrode Fz re-referenced to C7, because recent work suggests that the FFR measured with electrodes placed close to the mastoids (which are used in the Cz-averaged mastoid configuration) may pick up neural activity generated peripherally in the auditory nerve [60]. Data were also re-referenced to the right mastoid to replicate the horizontal montage used by Galbraith [53]—for this analysis, thirty subjects with good quality data and mastoids with similar noise levels on each side were included.

#### Stimulus presentation

A single missing fundamental complex tone was selected from the behavioural task for the EEG recording. A 120 ms long (48.8kHz sampling frequency) version with an absent fundamental frequency of 98Hz and energy only in the 8^th^, 9^h^, and 10^th^ harmonics (784Hz, 882Hz, 980Hz) was prepared using Matlab custom scripts, as was a 'fundamental present' (FP) equivalent that included power at the fundamental frequency (see Fig 1(B)). The MF version has the same pitch (to f0 listeners) but is perceptually distinct [53].

Auditory stimuli were delivered binaurally via insert earphones (ER3, Etymotic Research, www.etymotic.com) using Matlab software interfaced with a signal processing system (RX6, Tucker-Davis Technologies, www.tdt.com). All subjects listened to a block of MF stimuli before the FP stimuli to increase the likelihood that SP listeners maintained their SP perception [42]. In both the MF and FP blocks, 2500 stimuli in alternating polarity were presented at 80dB SPL. Blocks were 8.5 minutes long and subject comfort and alertness was checked informally between blocks.

#### EEG preprocessing

Data analysis was performed using EEGLAB [61] and custom Matlab scripts. Data were band pass filtered (80–2000 Hz for f0 analysis and 2-2000Hz for gamma analysis, using a 4^th^ order zero-phase Butterworth filter as implemented in EEGLAB's 'pop_basicfilter' function), epoched (-60 to 140 ms relative to stimulus onset), and DC correction was applied to the baseline period. Epochs containing myogenic or other physiological artifacts were removed by calculating the maximum absolute amplitude for each epoch and excluding the top 15% from each condition and polarity. This method serves to retain equal numbers of epochs for each subject and condition despite differences in baseline EEG amplitude as well as remove movement artifact, as confirmed by visual inspection. The mean minimum number of remaining epochs for each subject and conditions was 2110 (SD = 3.9). The spectra of the FFR portion of the averaged summation waves were obtained by phase-locking value (PLV) analysis using custom scripts, which provides comparable results to and is highly correlated with spectral amplitude methods but is more statistically sensitive [62]. For each subject and condition, a set of 400 epochs from the total pool of epochs was selected randomly with replacement. Each was trimmed to the FFR period, windowed (5ms raised cosine) to limit spectral splatter, zero-padded to 1s to allow for a 1Hz frequency resolution, and the phase of each epoch was calculated by discrete Fourier transform. The phase locking value for each epoch was computed by normalizing the complex discrete Fourier transform by its own magnitude, and averaging across 1000 iterations. From each subject and condition average, the mean f0 strength was taken (see 'Appendix: analysis methods' item 5, in [62] for formulae). The PLVs of the f0 and harmonics were obtained automatically using a script that selects the peak within a +/-7 Hz window around the corresponding peak in the stimulus and calculates the average PLV from a 5-Hz window centred on the peak; all subjects had a clear peak close to f0. PLVs have been shown to be highly correlated with measures of spectral amplitude for fundamental present stimuli [62]. To ensure that this relationship holds for missing fundamental stimuli and that PLV is an appropriate method for our research questions, and for comparison with previous work, we calculated the spectral amplitude of the time-domain summation wave by FFT (10 ms raised cosine ramp, zero-padded to 1s) for each subject and condition and assessed the degree of correlation between PLV and spectral amplitude at f0.

We used bootstrapping to statistically evaluate intraindividual differences between conditions (hypotheses ii) and iv)) [63]. We pooled epochs from both conditions and drew two samples randomly with replacement from them. Each sample matched the total number of epochs collected for a single condition. We calculated the difference in PLV at the fundamental frequency for each sample. We then repeated this procedure 1000 times per subject, to form a null distribution, and tested whether the difference in mean PLV from the correctly assigned epoch sets, resampled to the full number of epochs, lay within the 2.5^th^ to 97.5^th^ percentile range (i.e. two-tailed distribution with alpha = 0.05). Difference waves were also made for each subject, by subtracting single polarity time domain averages (positive–negative polarity averages). Spectral amplitude was calculated as described above to evaluate whether spectral energy at f0 might be observable and might differentiate conditions (see Results—Effect of presence of f0 energy in the stimulus).

#### Data analysis

We calculated Spearman's rho (r_s_) to assess rank correlations between perceptual bias and measures of musicianship, and current age. To test hypothesis i), that f0 strength is positively correlated with the subject's propensity to hear in the fundamental mode, we calculated Spearman's rho between f0 strength in the MF condition and perceptual bias, controlling for age, which is known to decrease FFR strength [64]. We also investigated the signed difference between f0 in the MF and FP conditions (e.g. MF–SP) vs. perceptual bias. Note that we could not evaluate what might appear to be the opposite relationship, that spectral listeners would have stronger harmonic representation, because the frequencies of the harmonics used in the study are too high to be strongly represented in the FFR (>700Hz). This means that the energy we observe at the second through 5^th^ harmonics cannot be assumed to be related to the strength with which the auditory system represents the physical properties of the stimulus.

To evaluate hypothesis ii), that the presence of f0 energy in the stimulus affects the FFR's f0 strength, we first compared the mean f0 PLV across stimulus conditions using a two-tailed Wilcoxon signed rank test, then evaluated within-subjects differences between f0 distributions generated by PLV in each condition, via resampling. The spectral amplitude of differences waves were visually inspected and f0 amplitudes statistically compared at the group level to rule out the possibility that interesting between-condition differences might have been removed by averaging over opposite polarities.

To test the secondary question that the mastoid-to-mastoid electrode montage reveals differences between MF and FP conditions, we calculated PLV as above, compared conditions using a Wilcoxon signed-rank test, and visually inspected each subjects' time and frequency domain data. We also calculated the amplitude spectra (i.e. by FFT rather than PLV) of single polarity averages, which is most similar to the analysis previously reported [53], to ensure that this methodological difference was not the cause of the observed results. To test the consequence of selecting one of the two electrode montages in current use, we calculated the same measures using the Cz to averaged mastoids montage. For comparison with cortical gamma-band results in Experiment 2, we repeated the PLV analysis using data that had been filtered to include lower frequencies (band-pass filter: 2-2000Hz), and calculated the mean PLV in the low gamma range (24–48 Hz) of the averaged spectrum in the MF condition. We assessed correlations between mean low gamma activity and perceptual bias, musicianship, and f0 strength.

### Results

#### Behavioural results

**Fig 2:**
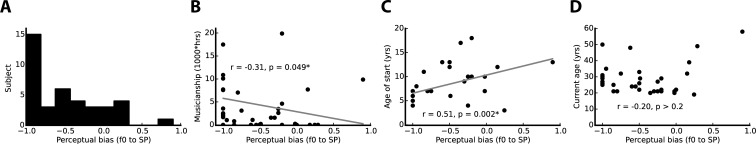
Perceptual bias and relationships to musical experience and age. (A) Distribution of perceptual bias in our sample (N = 39); this is weighted towards fundamental listeners but nonetheless includes people with a range of responses. (B) Perceptual bias was correlated with cumulative hours of musical training, and was negatively correlated with (C) the age of training onset in subjects who reported musical experience (N = 30). (D) Perceptual bias was not significantly correlated with current age.

The mean score on the perceptual bias task was -0.56 (range: -1.0 to 0.9); see Fig 2(A). Total hours of vocal and instrumental music practice was negatively correlated with perceptual bias (i.e. as previously reported, musicians were more likely to have fundamental responses; one-tailed r_s_ = -0.31, p = 0.049). Age of start of musical training among people reporting musical experience (N = 30) was also correlated with perceptual bias (one-tailed r_s_ = 0.51, p = 0.002; an earlier training start increased the likeliness of fundamental bias). We did not find a significant relationship between perceptual bias and age (two-tailed r_s_ = -0.20, p > 0.2).

#### Comparison of PLV and spectral amplitude at f0

The PLVs and spectral amplitudes at f0 were highly correlated in both conditions (FP: r_s_ = 0.80, p = 2.7e-08; MF: r_s_ = 0.82, p = 7.5e-09), confirming that the two measures contain shared information both in the responses to fundamental present responses as has already been shown [62], and in missing fundamental responses used here.

#### Variability in FFR waveform

**Fig 3:**
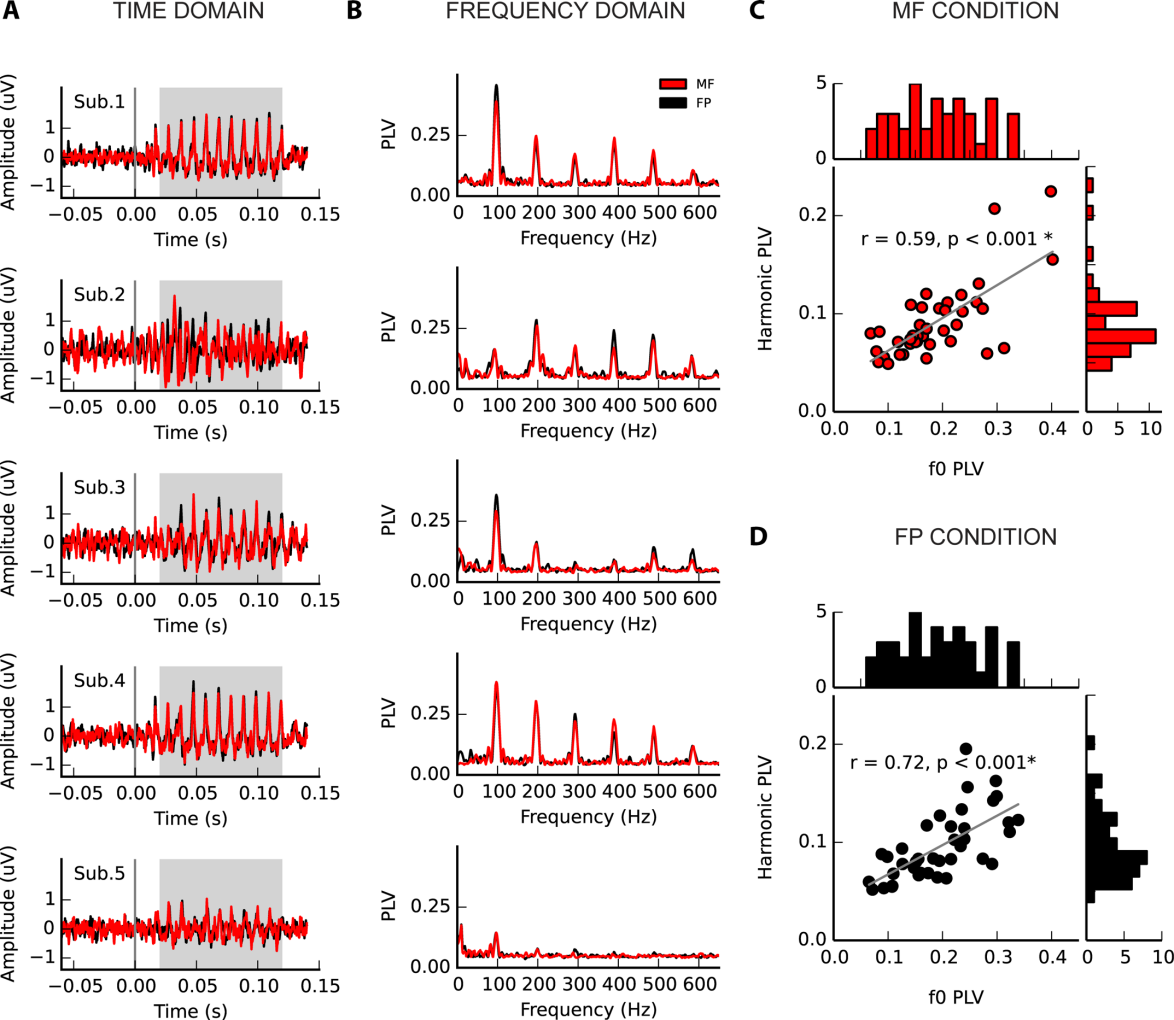
Inter-individual variability in frequency following response to complex tones that contain (black), or lack (red) energy at the fundamental frequency (f0). (A) Time domain representation of five example subjects (band-pass filtered between 80 and 2000Hz), demonstrating variation in ABR waveform. Grey shading indicates the FFR portion of the signal. (B) Frequency domain representation of the example subjects’ FFR. Variability is evident in the strength of the encoding of the f0 (98Hz) and its harmonics (integer multiples), and in their pattern of relative strength. The relationship between the f0 and mean harmonic PLVs (2nd-5th harmonics), in the (C) missing fundamental and (D) fundamental present conditions demonstrates considerable variability in the strength of each and their relationship, although in general people with a strong representation of the f0 have a strong representation of the harmonics. Histograms are associated with each axis to illustrate the distributions (above: f0 PLVs; right: mean harmonic PLVs).

Five subjects were selected to illustrate FFR variability; these are presented in Fig 3A and 3B. Considerable variability is present in the amplitude of the fundamental; for example, subjects 1, 3 and 4 have strong f0 representations, whereas 2 and 5 do not. Variability is also observed in the amplitude of the harmonics, as well as the relationship between the f0 and harmonic amplitude; for example, subject 4 has both a strong f0 and harmonic representation and subject 5 has both a weak f0 and harmonic amplitude, whereas subjects 1 and 3 are dominated by f0 and variable harmonics. Subject 2 instead has harmonics that are notably stronger than the f0. The variability in f0 PLV and mean harmonic PLVs (2^nd^–5^th^ harmonics) in the entire sample is presented in Fig 3(C) and 3(D). Subjects with stronger f0s tend to have stronger harmonics (FP: r_s_ = 0.72, p < 0.001; MF: r_s_ = 0.59, p < 0.001), though variability is evident in both the strength of the fundamental and the harmonics, and their relationship.

#### Relationship between perceptual bias and FFR f0

**Fig 4:**
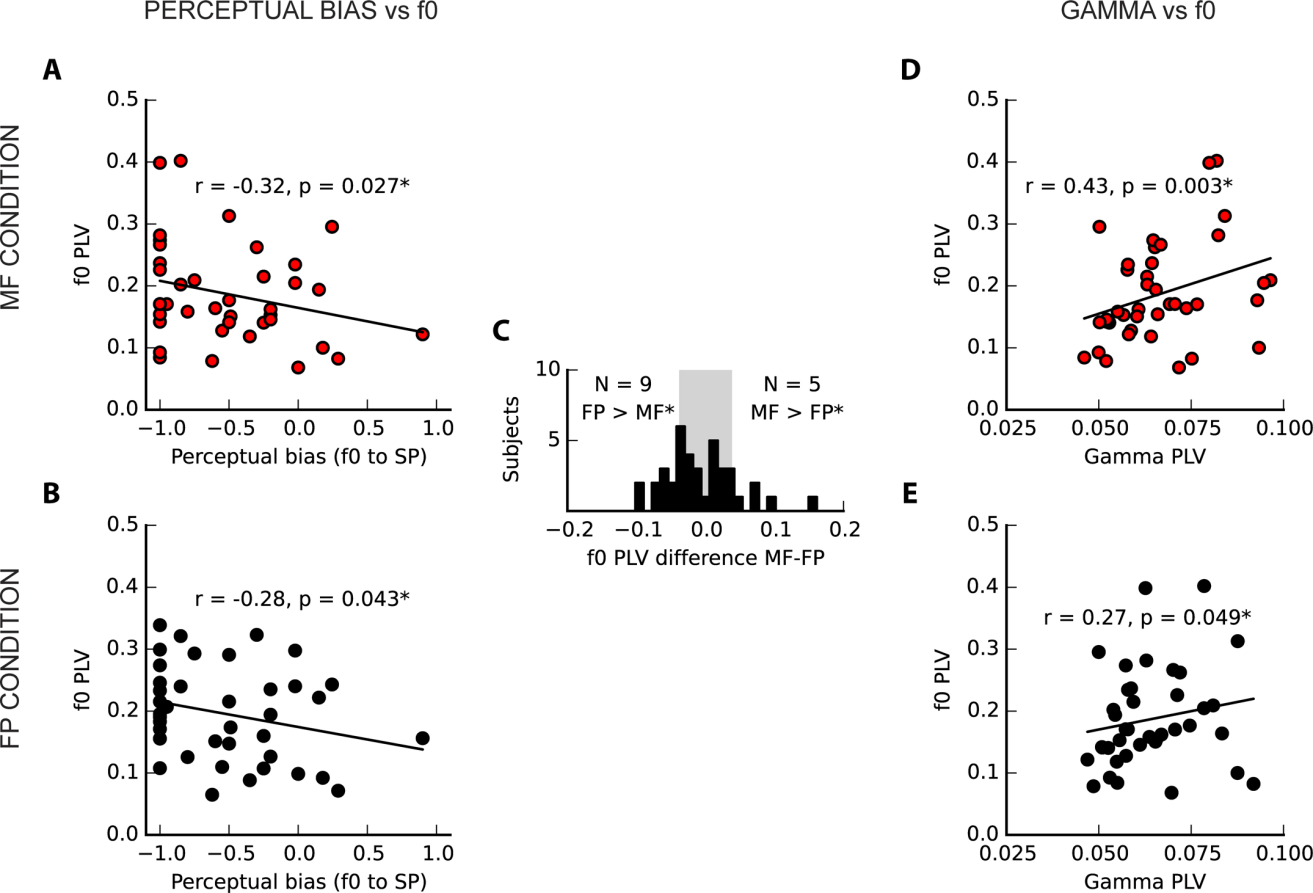
Experiment 1 results. (A, B) Fundamental perception bias was correlated with f0 peak magnitude in response to missing fundamental and fundamental present tones in each condition (the effect of age is controlled). (C) Histogram of PLV differences at f0 between conditions. While many subjects showed a statistically significant distinction between conditions as determined by resampling (14/39 subjects), subjects did not show consistency in the condition with greater amplitude; 9 had a greater amplitude in the FP condition and 5 had a greater amplitude in the MF condition. (D, E) The PLV of the f0 in each condition was positively correlated with the mean PLV in the low gamma frequency range in both conditions. Both the relationships between f0 PLV and perceptual bias and between f0 PLV and gamma PLV appear more clearly in the MF condition (red), when the fundamental frequency is not present in the stimulus and must be computed. FP: fundamental present condition; MF: missing fundamental condition.

The main hypothesis, concerning a relationship between perceptual bias and f0 strength in the MF condition, was supported (partial rank correlation, r_s_ = -0.32, p = 0.027; Fig 4(A)). A similar pattern was observed in the FP condition (partial rank correlation, r_s_ = -0.28, p = 0.04; Fig 4(B)). No trend was observed in the signed difference in f0 strength between conditions vs. perceptual bias (r_s_ = 0.02, p = 0.91).

#### Effect of presence of f0 energy in the stimulus

There was no effect at the group level of stimulus condition on the strength of the f0 representation (FP mean = 0.20, SD = 0.08; MF mean = 0.19, SD = 0.08; Z = 1.41, p = 0.16). At the individual level, 14 out of 39 subjects showed significant differences (in either direction) between conditions using a resampling approach (p < 0.05; Fig 4(C)); of those, 9 had a greater PLV in the FP condition and 5 had a greater PLV in the MF condition.

The spectral amplitude of the difference wave (positive—negative polarity) averages showed no distinct peaks at f0, and mean amplitudes at f0 were very small in both conditions (< 0.1). There was no significant difference between conditions, across subjects (Z = 0.49, p = 0.63).

#### Other sources of inter-individual variability

Hours of musical training demonstrated non-significant trends in the expected direction, with more hours of musical experience predicting larger f0 PLVs (FP: one-tailed r_s_ = 0.22, p = 0.09; MF: r_s_ = 0.14, p = 0.20). Start ages amongst musicians also showed non-significant trends in the expected direction (i.e. larger f0 PLVs for earlier training initiation; FP: r_s_ = -0.20, p = 0.14; MF: r_s_ = -0.25, p = 0.09). The signed difference between conditions was not clearly related to hours of musical training (r_s_ = -0.22, p = 0.17) nor start age (r_s_ = -0.15, p = 0.43). Age was not significantly related to f0 PLV in either condition, though the trend was in the expected direction in both conditions (FP: r_s_ = -0.17, p = 0.15; MF: r_s_ = -0.19, p = 0.11).

#### Effect of montage selection

f0 PLV when measured with the Fz-C7 montages was significantly correlated with f0 PLV when measured using the Cz-average mastoid montages, however, not perfectly so (FP: r_s_ = 0.69, p < 0.001; MF: r_s_ = 0.57, p < 0.001). As with the Fz-C7 montage, no effects of condition were found at the group level (Z = 1.02, p = 0.31). Only 21 subjects showed the same direction of difference across montages (i.e. either FP > MF or MF > FP). The relationship between f0 PLV and perceptual bias observed using the Fz-C7 montage was not evident in the Cz-averaged mastoids montage in the MF condition (r_s_ = 0.05, p = 0.62), nor in the FP condition (FP: r_s_ = 0.01, p = 0.52).

There was very little signal in the data using the horizontal montage as compared with the other montages (FP mean f0: 0.11, SD = 0.13; MF: mean f0: 0.11, SD = 0.12), and the two conditions were not statistically distinguishable (Wilcoxon signed rank test: Z = -1.21, p = 0.89). We also calculated the spectral amplitudes of only a single polarity average, as was used in previous work [53]; f0 amplitude was very small and was not greater in the FP condition than in the MF condition (FP: mean = 0.02uV, SD = 0.001; MF: 0.04, SD = 0.001).

#### Low gamma band activity

Mean gamma band activity in the MF condition was not correlated with perceptual bias (r_s_ = -0.02, p = 0.91), nor hours of musicianship (r_s_ = -0.04, p = 0.81), but it was significantly correlated with f0 PLV, more clearly in the MF condition (MF: r_s_ = 0.43, p = 0.003, FP: r_s_ = 0.27, p = 0.049).

### Discussion

#### Perceptual bias is related to musicianship

Our results demonstrate a relationship between perceptual bias and measures of musicianship, such that musicians with more training and an earlier start age were more likely to have a fundamental bias. This finding supports previous work [35], and indicates that despite a fundamental-skew in the perceptual mode bias measure, the range of stimuli used was sufficient to capture individual differences in auditory perception. We confirmed and quantified the variability in FFR f0 and harmonic representation in the frequency domain.

The discrepancy in the distribution of perceptual bias scores with a previous large-scale study that found a more symmetrical bimodal distribution is most likely due to differences of approach [33]. That study included an additional 'fundamental present' condition in behavioural testing that allows octave-shifted responses (i.e. perceiving the 2^nd^ harmonic as the pitch of the tone) to be accounted for and excluded from the overall perceptual bias score. Studies that have not used this approach yield similar distributions of perceptual bias as the present study (e.g. [31,35]). Including octave-shifted perceptual responses did not qualitatively change the main findings of a relative hemispheric lateralization in the previous work [33], and similarly we are able to observe the effect of interest, here. However, future work could include an octave-shift control condition to allow for assessment of this effect.

#### Individual differences in FFR f0 strength are correlated with perceptual bias

As hypothesized, the strength of the f0 in the MF condition was correlated with perceptual bias, with fundamental listeners more likely to have stronger f0 representations. There was no difference at the group level in the FFR f0 PLV in f0 response to stimuli with the fundamental present or absent. This could indicate that however the energy physically present at the stimulus' f0 is encoded and perceived, it is not measured as part of the FFR; however, at the individual level, distinctions could in fact be made between the conditions for many subjects (36%), suggesting that the specifics of the stimulus are captured in the FFR, but not in a consistent fashion across participants.

#### Electrode montage affects FFR f0 strength

We attempted to replicate the finding that an FFR recorded using a horizontal electrode montage would show f0 energy in the FP but not MF conditions [53], while f0 energy was found in both conditions using a vertical (Cz to mastoids or earlobes) montage, as we have been observing. This work has been interpreted as demonstrating that the f0 representation of MF stimuli appears in the rostral brainstem [2,53]. We did not find evidence that the f0 is stronger in the FP condition than the MF condition; in fact the signal amplitude in both conditions is very small and inconsistently observed between subjects. One possible explanation is that the previous work used monaural stimulation, and it has been shown that electrodes placed at the mastoid or earlobe pick up activity from the auditory nerve, which does show activity related to amplitude modulation in the signal [60,65]; this auditory nerve activity might be cancelled out in our binaural configuration when the horizontal montage is used. We do however find support for the suggestion that different electrode montages measure different events [53]. Comparing the two main electrode montages in current use, Cz-average mastoids and and Fz-C7, we found that while the f0 amplitudes are significantly related, variability is also demonstrated, and behavioural relationships that we identified using the Fz-C7 montage were not all replicated in the second montage. Only 54% of subjects showed consistency in the direction of the difference between f0 in each condition. This suggests differences in the relative contribution of sources, with two implications: the two montages may not be fully interchangeable, and that differences in individual anatomy such as head shape might contribute to inter-individual variability in the strength of the recorded signal, as well as possibly contribute to some of the complex relationships to behaviour that have been observed. This finding underscores the importance of methods which allow source separation [60] such as MEG [18].

#### Conclusion, limitations, and further questions

We can conclude that a person's general perceptual bias, and therefore the cognitive machinery that supports pitch perception, contributes to variability in the FFR's f0 strength and is measurable in it. However, we are not able to confirm that subjects were hearing in the spectral or fundamental mode during the FFR recording using this design. Because the auditory context [36] as well as repetition [38] can influence perception, and because responses to specific stimuli are variable [31], it is possible that at least some spectral listeners were hearing in the fundamental mode during the FFR recording. The observed relationship between f0 PLV in the MF condition and general perceptual bias might be attributed to relatively permanent differences between people due to biology or long-term training. It is also possible that the effect of fundamental vs. spectral perception acts more flexibly, in an online top-down manner, which provides the motivation for the second experiment.

## Experiment 2

To be able to specifically examine the neural correlates of perceptual mode in a highly controlled fashion and thereby further support the hypothesis that f0 variability in the FFR is related to pitch perception, we developed a sensitive within-subject paradigm that allowed perceptual mode to be brought under voluntary control. This was made possible by first selecting harmonic numbers for each subject within an ambiguous zone between their thresholds for fundamental and spectral perception, creating custom MF melodies based on these harmonic numbers, and then using pure-tone primes to help the subject select and attend to each perceptual mode within the melody. We first assessed if subjects can reversibly switch between perceptual modes using behavioral data. We then recorded FFRs while subjects listened to identical stimuli in either the fundamental or spectral modes, and evaluated whether the strength of the f0 representation when subjects were trying to hear the f0 was related to their behavioural performance. Our goal was to extend the finding in Experiment 1, in which we found a correlation between f0 representation in the MF condition and perceptual bias, by actively causing f0 modulation with an experimental manipulation. Because an increase in cortical low-gamma band activity has been reported following a spectral-to-fundamental perceptual shift [38], we also assessed whether low-gamma band activity was greater in the f0-listening condition. Finally, we looked at the relationship between the neural correlates of mode perception and musicianship. This design served to control for possible effects of diminishing or fluctuating attention [28] in the first experiment, as conditions could be interleaved and active responses to periodic mode-specific mismatched stimuli were tracked.

### Methods and Materials

#### Participants

35 subjects who were 40 years old or younger were recruited. Five subjects were excluded because bistable harmonics could not be found for them within the testing range, and one additional subject was excluded due to a technical problem. The mean age of the remaining 29 subjects was 24.5 (SD = 4.9); 14 were female; 26 were right-handed, and 3 reported ambidexterity. All subjects reported having normal hearing and no neurological conditions. Data about musical history were collected via an online survey (Montreal Music History Questionnaire, [57]). The mean total instrumental and vocal practice and lesson hours was 4278.2 (SD = 6213.2), and subjects with musical experience started training from 3 to 17 years (mean = 8.8, SD = 4.5). One subject was a native tonal language speaker. Written informed consent was obtained and experimental procedures were approved by the Montreal Neurological Institute Research Ethics Board and the Research Ethics Committee of the Faculty for Arts and Sciences of the University of Montreal.

#### Study design

Subjects completed the MMHQ before the experiment. Prior to the EEG session, subjects performed a set of behavioural tasks (~15 mins). These were designed to: ensure the subject had normal melody perception for the frequencies used to test perceptual mode switching, ensure the subject could hear each of the perceptual modalities within the range of series of harmonic numbers used in later parts of the experiment, confirm that the subject could reliably switch between perceptual modes in their custom harmonic ranges, and obtain a measure of perceptual mode hearing accuracy. Prior to the FFR recording, subjects were familiarized with the perceptual task and their subjective experience of being able to successfully switch between perceptual modes was confirmed. During the recording, subjects were asked to identify deviant tones in each listening mode to control for attention. Following the FFR recording, we recorded click-FFRs as in Experiment 1 to screen out auditory system pathology, and subjects were asked to complete a short survey capturing their subjective experiences.

#### Ambiguous harmonic determination and testing

Behavioural testing took place in the acoustically and magnetically shielded booth used for EEG testing, using insert earphones (ER3, Etymotic Research, www.etymotic.com), with auditory stimuli (sampling frequency = 48828Hz) that were produced and presented using custom Matlab scripts.

**Fig 5:**
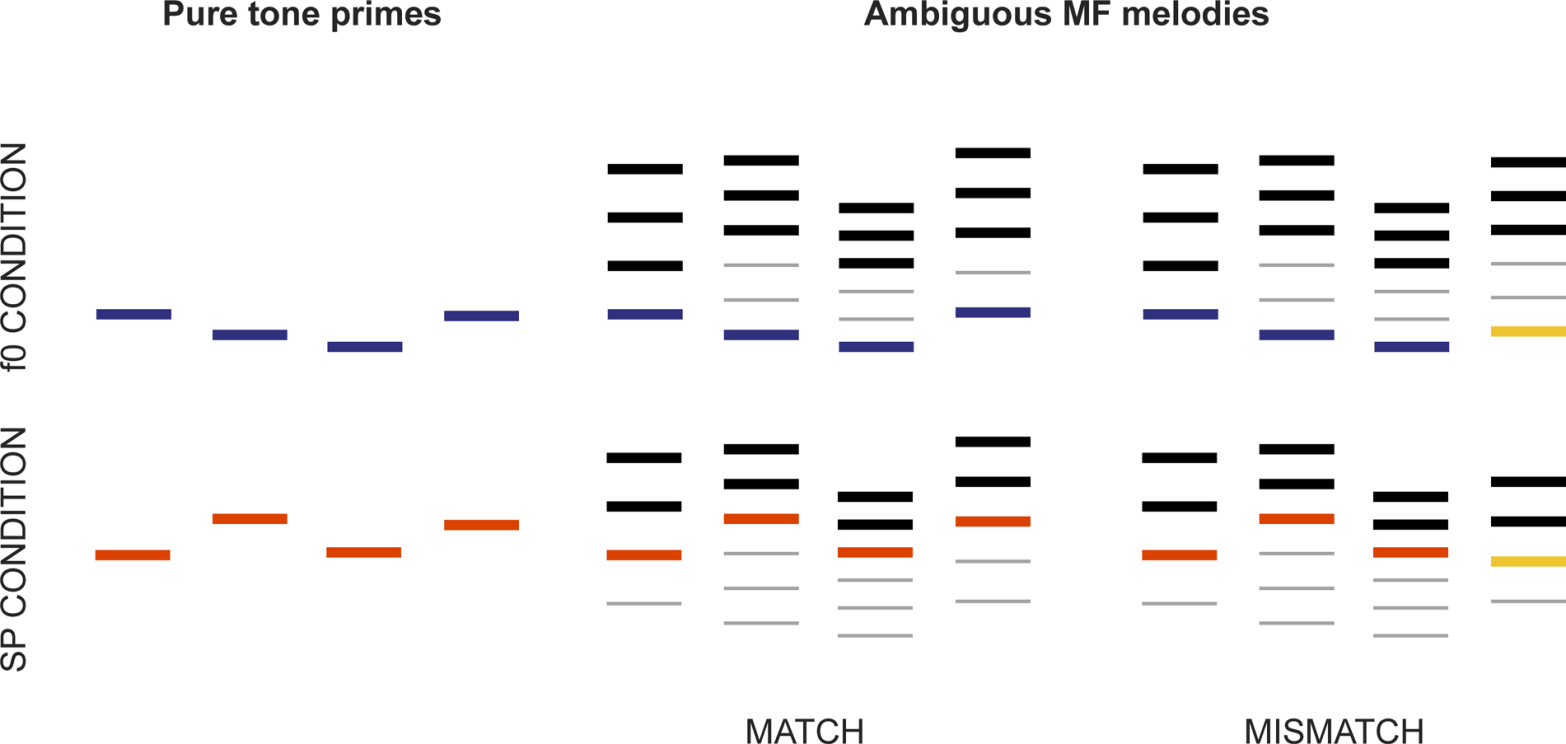
Experiment 2 task design. In behavioural testing, subjects heard pure tone primes that matched the pitches of a spectral or fundamental perception of subsequently presented complex harmonic melodies, and were asked whether there was a match or mismatch between the two. This was used to determine the harmonic range used for each subject in the ABR recording (see Fig 6), and confirm that subjects could hear in both perceptual modes. During the ABR recording, subjects were primed to hear in a single repeated melody in their specific harmonic range, and were asked to identify occasional mismatches to control for attention and perception.

i) Melody perception (control): Subjects were presented with 5 trials, each consisting of a pair of identical or non-identical melodies. Each melody was comprised of 4 pure tones of 160 ms (10 ms raised cosine ramps) separated by a silence of 300 ms, with a 2300 ms gap between pairs of melodies (similar to Fig 5, except that both primes and targets used pure tones). Sine tones were created based on a 200 Hz central frequency (close to G3 of 196hz on the Western musical scale), one full tone higher (224.49 Hz, ~A3), and two full tones lower (178.18 Hz, ~F3; 158.74 Hz, ~D#), permuted randomly into 4-tone melodies. These frequencies were central to the experiment as they corresponded with MF frequencies throughout subsequent testing. Each subject heard the same pseudo-randomized set of trials in the same order. After each trial, they were asked to enter a 'same' or 'different' judgement into a computer.

ii) Harmonic number range determination: Subjects were presented with trials similar to those used in i), except that only the first melody was made from pure sine tones; this was used to prime either spectral (SP) or fundamental (f0) mode perception in the second melody, which consisted of 4 missing fundamental complex tones (illustrated in Fig 5), each comprised of three consecutive cosine-phase harmonics. As above, harmonic complex tones were 160 ms long with 10 ms raised cosine ramps, and were separated by 300 ms silent gaps.

To conform to subjects' Western scale musical expectations, we held the interval between notes equal to one full tone. For this to be possible and to create melodies that could not be matched using the opposite perceptual mode purely on their pitch contour, it was therefore necessary to use different sets of consecutive harmonic triples for each note (e.g. note 1: 2^nd^–4^th^, note 2: 4^th^–6^th^, note 3: 4^th^–6^th^, note 4: 3^rd^–5^th^). The f0 frequencies in all parts of the experiments were therefore the same for each participant, whereas the harmonic numbers and corresponding frequencies differed. The frequencies of the lowest and highest harmonic partials used in this test (2^nd^–32^nd^) ranged from 448.98 Hz to 5714.64 Hz. The melodic contours were held constant across subjects.

**Fig 6:**
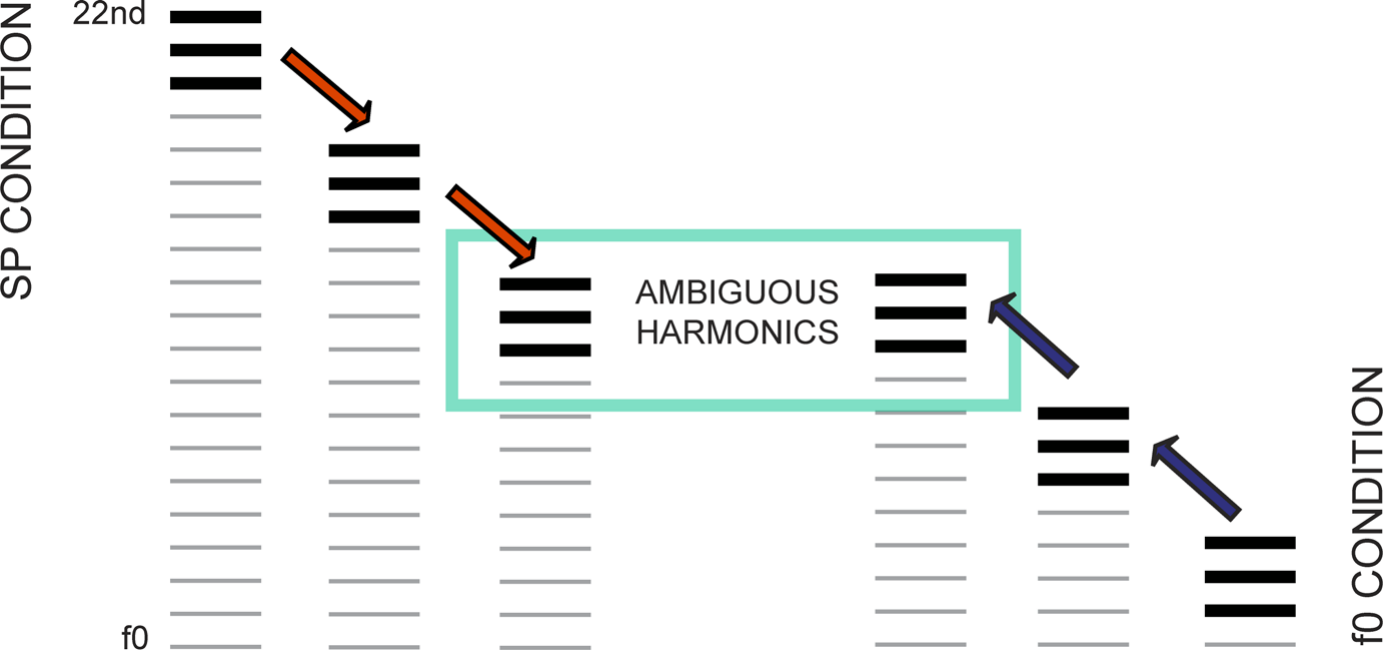
Harmonic number range determination. Using the task illustrated in Fig 5, a range of ambiguous harmonics for each subject was identified: first, MF complex tones with very high harmonics, which encourage spectral perception, were presented. Subjects’ match-mismatch identification accuracy was evaluated, and the harmonics decreased in steps of 4 until a threshold of spectral perception was found (i.e. fewer than 4/5 trials correct) or the range was exhausted. Second, complex tones with low harmonics, which encourage fundamental perception, were presented, and increased according to accuracy until a threshold was found. A range of harmonics based on the midpoint between the thresholds was used for further behavioural testing to confirm that each subject could hear in both modes, and melodies based on the central harmonic were created for the ABR recording.

Harmonic complex melodies matched pure tone primes 50% of the time (randomized order). The test was divided into two parts: SP and f0 perceptual modes, which were each subdivided into blocks of 5 trials. Before each block, two unscored practice trials were presented without feedback. These were matches, which are easier than identifying mismatches when perception is ambiguous, and served to help the subject listen in the correct mode on the subsequent test trials. Accuracy was assessed at the end of each block, which either ended in the case of poor performance (3 or fewer correct out of 5) or the harmonic number was adjusted by 4 for an additional block (e.g. from 12 to 8), until accuracy decreased below criterion or the range was exhausted; the harmonic number of this final block was taken as the limit for that perceptual mode. We first presented harmonic triplets at the top of the available range, because partials with higher harmonic numbers favour SP mode perception [33], and accurate performance resulted in a downwards adjustment of harmonic numbers for the following block (Fig 6) until the limit was found. We then presented harmonic triplets in the lowest available range, which encourages fundamental perception, and progressively raised them until the limit was reached. The subjects' optimally bistable or ambiguous harmonic was selected as the midpoint between their SP and f0 perceptual limits.

iii) Confirmation of perceptual switching. To confirm that subjects were able to consistently hear in both perceptual modes at their optimally bistable harmonic as determined in ii), and to measure their relative ability to do so, we generated an additional set of 30 match-mismatch trials as described in ii), half in each mode, that was centred on the subjects' ambiguous harmonic numbers as determined in ii). Starting with the SP condition, 5 trials of each mode were presented at the optimal central harmonic, and 5 of each mode were presented one harmonic number above and one below each listener's optimally ambiguous harmonic. Each mode block started with two unscored practice trials to help the subject listen in the correct mode. All stimulus and trial variables were as described above; please see 'Comparison of behavioural task and EEG recording stimuli' below for a description of the differences between the behavioural testing described here and the stimuli used for the EEG recording). The number of correctly identified matches and mismatches was used to generate a percent accuracy score.

#### Subject preparation and EEG data acquisition

Recordings were conducted as described in Experiment 1, with the addition of two electrodes at C3 and C4 (10–20 International System) in order to examine gamma activity in each hemisphere.

#### Stimuli used for EEG data acquisition

Customized missing fundamental melodies for each subject were prepared using Matlab scripts, using harmonics centred on each subject's optimally ambiguous harmonic as determined above. Stimuli used during the EEG recording had the same properties as those used in the harmonic number range determination and confirmation of perceptual switching tasks, except that in contrast to the behavioural task stimuli which used 4 different tones, the melodies for the EEG recording repeated one of the tones (notes 2 and 4; f0 = 158.74Hz). This tone was analyzed to calculate the FFR f0 and gamma band activity, and repeating it served to limit the length of the recording. The target harmonic complex was presented ~1580 times per subject per condition.

For SP block deviants, the harmonic numbers were increased such that the SP perception increased by a tone, but the f0 perception remained unchanged; for f0 block deviants, the space between the harmonics was increased but the frequency of the lowest present partial remained the same, such that the SP perception was steady but the f0 perception increased by a tone. Because piloting revealed that deviants from the unattended mode remain somewhat perceptible to many subjects, we used only deviants in the same mode as a block. Auditory stimuli were delivered as described in Experiment 1, except that complex harmonic stimuli were presented at 70dB SPL because the higher frequencies used in this experiment were experienced as uncomfortably loud by some subjects at higher sound levels.

#### Stimulus presentation

The customized missing fundamental melodies were presented in alternating positive and negative polarity in a continuous stream with a 460 ms gap between them (i.e. 1-beat) such that each melody was 2300 ms in length. The experiment was broken into 12 blocks (5.5 minutes each) of 145 melody presentations, of alternating perceptual modality.

Each block started with 3 repetitions of the pure tone prime melody (either SP or f0) to prepare the subject to listen in the appropriate perceptual mode. Subjects were active participants in the perceptual process and reported spontaneously using a variety of mental strategies in order to maintain the correct perception, which is aided but not passively determined by the pure tone primes. Five additional pure tone prime melodies were inserted pseudo-randomly into the complex harmonic melody stream as memory aids, and 5 deviant melodies were also included to control for attention and confirm that subjects continued to hear in each perceptual mode during the experiment. Subjects responded to deviant tones with a button press. Responses were considered 'hits' if they occurred within the 2300 ms following their presentation, and total accuracy scores were calculated for each condition.

#### Comparison of behavioural task and EEG recording stimuli

For the purposes of confirming normal melody perception (i), determining the subject's optimal amibguous harmonic (ii), and confirming that they could hear in both perceptual modes (iii), we used a match-mismatch design in which subjects were presented with two melodies and were asked to make a judgement, which triggered the next trial. To obtain a range of responses, subjects received a pseudo-randomly created set of melodies. Two practice trials were used per block to help the subject listen in the appropriate mode, which was also facilitated by every trial being preceded by a pure tone prime as a target.

For the purposes of EEG recording, a single custom missing fundamental melody very similar to those used in the behavioural design was repeated multiple times in succession; no judgement (other than identification of the occasional deviants) was required. This helped subjects stay in the appropriate perceptual mode for several minutes at a time given several initial primes and randomized reminders, and allowed for collection of enough epochs for FFR analysis within a single experimental session.

#### Post-recording survey

We attempted to capture subjects' phenomenological experiences in a brief written post-experiment survey, followed by a short informal discussion in which subjects were encouraged to share their impressions. These strategies were included based on reports from pilot subjects. The survey asked for ratings on a 10 point Likert scale: 1) For how much of the experiment did you feel awake and alert? 2) How hard was it for you to concentrate on the right melody? 3) For you, was it easier to concentrate on: the high pitch melody, the low pitch melody, or both were equally salient (select one). 4) When you were able to concentrate on the correct melody, did you feel as if you could still hear the other melody? (yes/no, if yes how often on a 10 point scale.) 5) Which strategies did you use? Check all that apply: A) Imaging the melody (singing it in your head), B) Creating a visual image of the melody (imagining the notes or a pattern going up and down), C) Focusing on the high or the low pitch, D) Pretending to play an instrument, or E) Other (please specify).

#### Data analysis

We tested hypotheses iii), that subjects can learn to reversibly switch between perceptual modes, by calculating the mean accuracy for the 30 melodic pairs close to the central harmonic frequency and comparing it to chance levels using one-tailed t-tests corrected for multiple comparisons.

We tested hypothesis iv), that f0 representation in the f0 listening condition is related to behavioural accuracy, by correlational analysis; and that f0 representation differs between listening conditions by bootstrap resampling (described in Experiment 1 methods) to assess whether responses to each condition differed for each subject. We also planned to evaluate relationships with the signed difference between conditions in addition to the amplitude in a single condition in case group-level single-amplitude differences were obscured by the custom stimuli, because the harmonic composition of stimuli affects the strength of the f0 [62]. EEG data were processed as described in Experiment 1 methods. We used the Cz-averaged mastoids for this analysis, because we observed that effort-related tension in facial muscles produced strong myogenic noise in the Fz channel in some subjects. We assessed secondary questions concerning a relationship between f0 measures and musicianship. Non-parametric rank correlations are used (Spearman's rho).

We then tested hypothesis v), that cortical low gamma band activity (24–48 Hz) is affected by condition, also using PLV methods as described in Experiment 1. For gamma analysis, previous work did not find a significant hemispheric asymmetry [38], although results in the right hemisphere were more consistent. We therefore focused on a single electrode, selecting C4 for its proximity to the right auditory cortex, which is specialized for aspects of tonal processing [51,66–71] and the strength of FFR f0 signal attributed to it is correlated with fine pitch discrimination ability [18]. However for completeness, we performed an equivalent analysis on the left hemisphere electrode (C3). We evaluated the rank correlations of between condition differences between f0 PLV and gamma at the same electrode, as well as their relationship to behavioural measures. The difference between conditions in each measure rather than the absolute value is used here because the research question concerns a within-subject change in neural representation across conditions, rather than the relationship between neural correlates of a relatively stable bias, as in experiment 1.

### Results

#### Behavioural results

**Fig 7:**
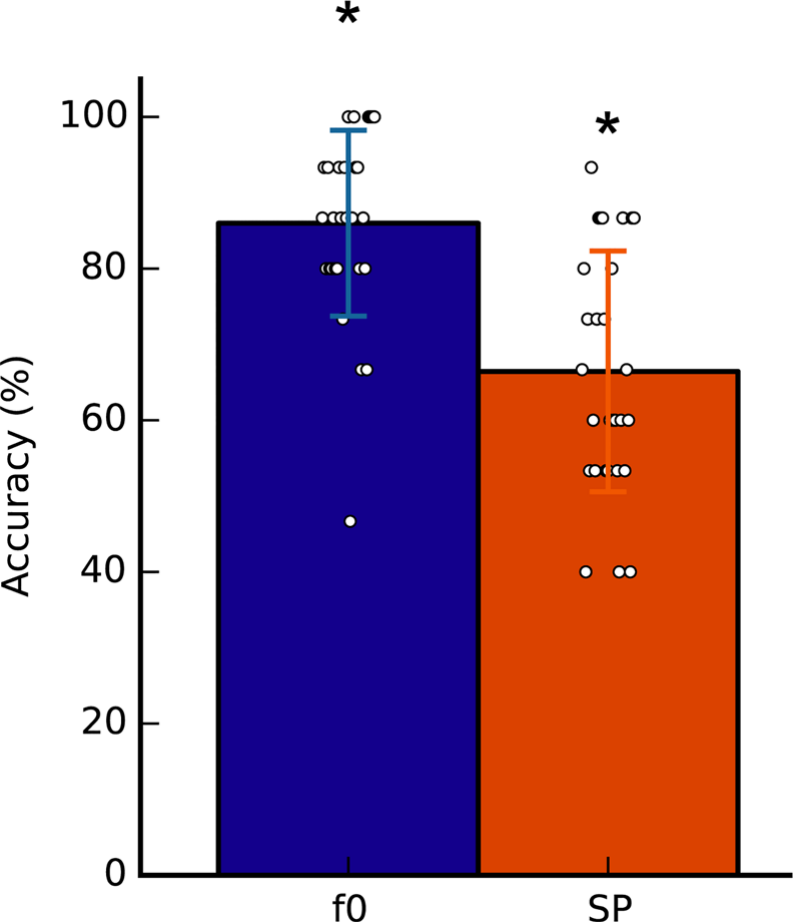
Experiment 2 behavioural results: accuracy identifying matches and mismatches in trials of each condition. Accuracy was significantly above chance in each listening condition. Subjects had significantly better scores in the f0 condition. Mean accuracy across conditions was used for further analysis. Error bars indicate +/-SEM; individual data are superimposed to illustrate the distribution.

1. Melody perception: all subjects achieved 100% accuracy, confirming normal simple melody processing.
2. Harmonic number range determination: the mean harmonic number selected was 16.7 (SD = 5.2), and the median and mode was 21 (numbers refer to harmonic numbers of the tone of interest for FFR analysis); this skew towards higher harmonics, which encourage spectral perception, is consistent with our finding of a generally fundamental bias in Experiment 1.
3. Confirmation of perceptual switching: the mean accuracy for the 30 melodic pairs close to the selected harmonic frequency was 76.2 (SD = 12.4), which was significantly above chance levels for each condition (Wilcoxon signed rank tests: f0: Z = -4.7, p < 0.001; SP: Z = -4.01 p < 0.001; Fig 7). Two subjects had chance-level accuracy (53%), these subjects were included because they reported being able to hear their custom melody in both modes immediately prior to the task, successfully identified deviants in both conditions during the task (85% and 95%), and reported that the task grew easier with practice. On average, subjects were less accurate in the spectral condition than the fundamental condition (f0: 86.0%, SD = 12.3; SP: 66.4%, SD = 15.9; Wilcoxon rank sum test: Z = 4.31, p < 0.001). The mean accuracy across both conditions was used for further analysis.

#### Deviance detection

During the EEG task, subjects were able to detect subtly altered deviant tones embedded in the melodies with high accuracy in both listening conditions: f0 condition mean 95.6% (SD = 7.7); SP: 94.4% (SD = 8.8); accuracy did not differ significantly between conditions (two-tailed Wilcoxon rank sum test: Z = 0.91, p = 0.36). This confirms that subjects were attending to the stimuli and continued to be able to hear in both perceptual modes throughout the EEG recording.

#### F0 representation in the FFR vs. perceptual condition

**Fig 8:**
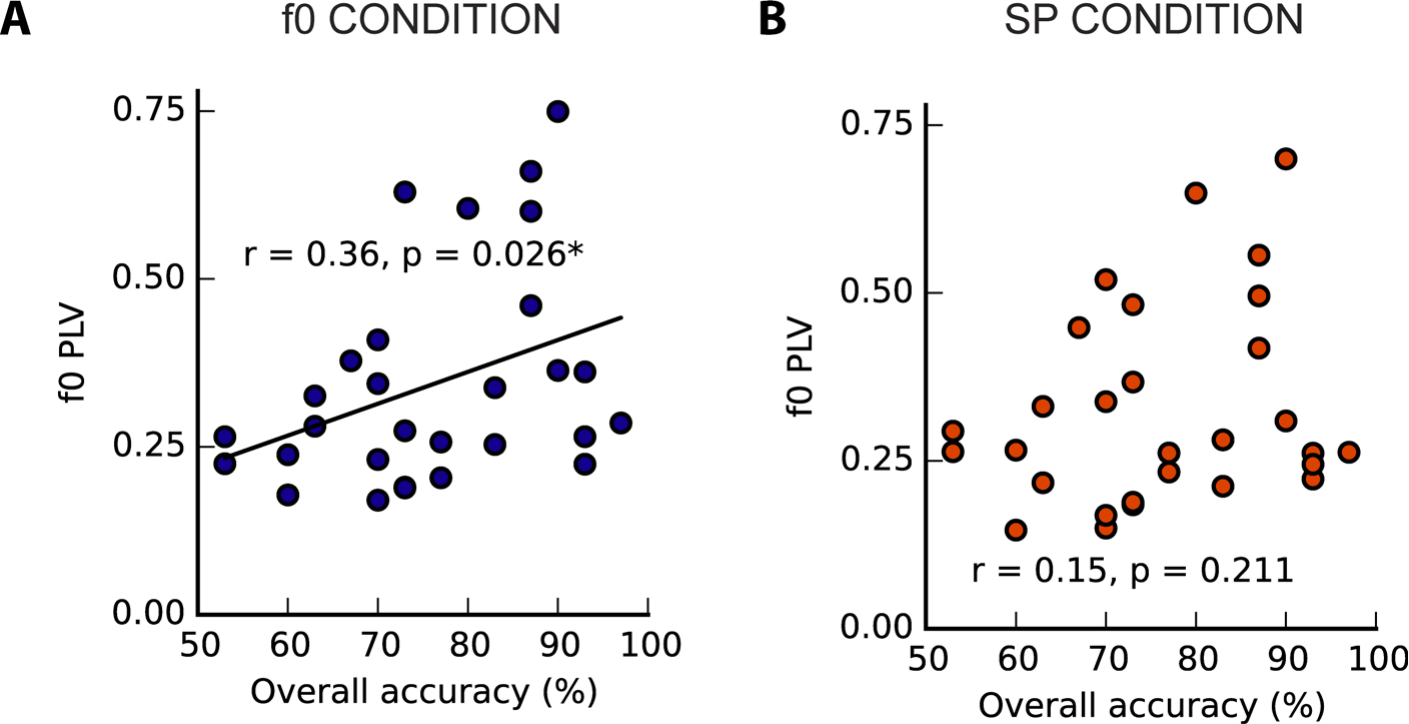
Accuracy on the listening mode task compared with peak f0 magnitude in the FFR. (A) In the fundamental listening condition, peak f0 magnitude correlated significantly with overall task accuracy, but in the spectral listening condition (B) the trend did not reach significance. The difference between the f0 peak magnitude in each condition (i.e. SP–f0) was also significantly correlated with task performance.

The strength of f0 representation in the f0-perception condition was positively correlated with overall perceptual accuracy (Fig 8(A); f0 condition: r = 0.36, p = 0.026). The trend between f0 PLV and perceptual accuracy in the SP listening condition was not significant (Fig 8(B); SP condition: r = 0.15, p = 0.211). The two correlations are not significantly different from one another (Fisher's z-test, two-tailed: z = -1.15, p = 0.25). The difference between PLVs (SP-f0) was significantly correlated with the perceptual task accuracy, such that a larger between-condition difference favouring the f0 perception was related to greater accuracy (r_s_ = -0.40, p = 0.015).

**Fig 9:**
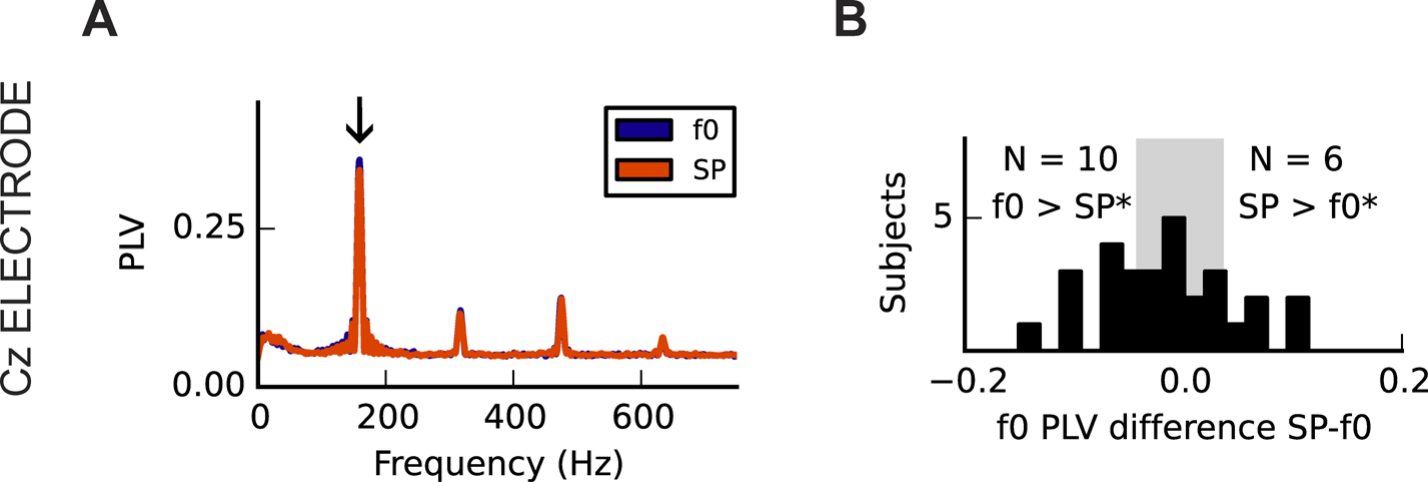
Neural correlates of perceptual condition to a complex harmonic stimulus at the fundamental frequency. (A) Phase-locking values averaged across subjects for each condition as a function of neural response frequency (Cz-averaged mastoids electrode channel) show no difference at the group level at f0 (arrow; 160Hz). However, (B) for many individuals (16/29), the f0 peak in each listening condition was statistically distinguishable in either the f0 > SP or SP > f0 direction, as calculated by using a resampling distribution.

The frequency-domain grand averages for each condition are illustrated in Fig 9(A), demonstrating that clear f0 representations can be elicited despite the wide range of harmonics used. The mean PLV at f0 was 0.34 (SD = 0.16) in the f0 listening condition and 0.33 (SD = 0.15) in the SP listening condition; this difference was not significant (two-tailed Wilcoxon rank sum test: Z = 0.42, p = 0.67). Both distributions were right-skewed, and the f0 amplitudes between conditions were significantly correlated (one-tailed r_s_ = 0.88, p < 0.001). Despite no unidirectional effect of condition at the group level, we observed significant differences in the f0 distributions at the individual level in 16 out of 29 subjects (Fig 9(B)).

We did not find a significant relationship between f0 PLV in separate conditions and hours of musical training (f0 condition: r_s_ = 0.17, p = 0.18; SP condition: r_s_ = 0.13, p = 0.25), nor was the difference in f0 amplitude at the Cz electrode related to hours of musical training (r_s_ = 0.02, p = 0.91).

#### Low gamma band activity

**Fig 10:**
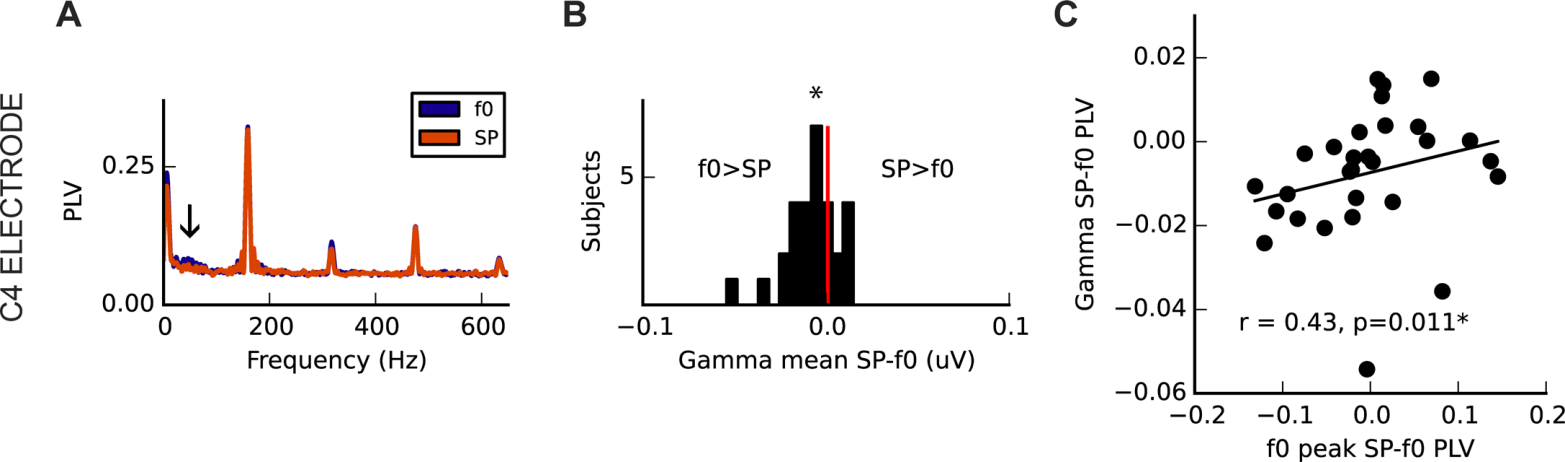
Neural correlates of perceptual condition to a complex harmonic stimulus in the low gamma band (24-48Hz) at an electrode placed over the right hemisphere (C4). (A) In spectra at electrode C4 (above right hemisphere), an average difference is observed in the gamma range (arrow). (B) Across subjects, gamma band amplitude was significantly greater in the f0 condition and (C) the difference between conditions in the FFR f0 and gamma band was significantly correlated. Relationships between gamma activity and the behavioural measures were not evident.

As predicted, the mean PLV of cortical gamma band activity over the right hemisphere was greater in the f0-perception condition than in the SP-perception condition (Fig 10A and 10B; Wilcoxon signed rank, one-tailed: Z = -2.54, p = 0.011). Although the difference in f0 PLV across conditions was correlated with the difference in gamma band activity across conditions at the same electrode (r_s_ = 0.43, p = 0.011; Fig 10(C)), there did not appear to be a direct relationship between gamma strength in each condition and perceptual switching accuracy (f0 condition: r_s_ = 0.12, p = 0.272; SP condition: r_s_ = 0.24, p = 0.101; SP-f0: r_s_ = -0.02, p = 0.46). Gamma PLV was not significantly related to hours of musical training (f0 condition: r_s_ = -0.12, p = 0.740; SP condition: r_s_ = -0.09, p = 0.680). As reported by earlier work [38], gamma activity did not differ significantly between magnetoencephalographic measurements taken over the left and right hemispheres, in the f0 condition (Z = 1.02, p = 0.304). A similar difference between conditions (Z = -2.11, p = 0.035) was observed at C3 as compared with C4, though the relationship between f0 PLV difference and gamma difference was non-significant (r_s_ = 0.20, p = 0.152).

#### Musicianship vs. perceptual accuracy

Across the entire sample, hours of musical training was not significantly related to accuracy on the perceptual switching task, though a trend was observed in that musical training was associated with better accuracy (r_s_ = 0.26, p = 0.08). Age at which musicians started training was not significantly correlated with perceptual switching accuracy (r_s_ = 0.10, p = 0.66).

#### Post-recording survey

Subjects reported feeling alert during the experiment (mean rating = 8.10, SD = 1.29, higher numbers indicate feeling alert more of the time), and that they were generally able to concentrate on the right melody (mean rating = 3.76, SD = 2.23, higher numbers indicate greater difficulty). 20 subjects reported that it was easier to concentrate on the f0 than SP melody, 2 reported that the SP was easier for them, and 7 believed both were similarly easy. When successfully concentrating on the correct melody, 7 subjects reported only hearing in one perceptual mode at a time, whereas 22 reported hearing in both perceptual modes simultaneously to some degree, an effect which they rated as occurring some of the time (mean rating = 6.95, SD = 2.08, where 10 indicates 'all the time'). Subjects reported a variety of strategies to help them listening the correct mode, with most (21 subjects) subjects using them in combination: 'singing in the head' = 18, visualization = 18, focusing on high or low pitch = 14, pretending to play an instrument = 5, other = 2, which included associating notes with syllables to create words. Several subjects commented to the researchers that the task became easier for them over the course of the experiment.

## General Discussion

### Individual differences in FFR f0 strength are correlated with perceptual bias

In Experiment 1, we presented complex harmonic tones that either included or omitted energy at the fundamental frequency, and evaluated the effect on the representation of f0 in the FFR. We confirmed the prediction that individual perceptual bias was related to the strength of f0 representation in their FFR. This finding shows that relatively stable MF perceptual biases [31] are paralleled not only by anatomical differences in cortical structures like Heschl's gyrus and in electrophysiological evoked responses of cortical origin [30,33], but also by differences in the FFR f0: a measure of fast temporal fluctuations related to basic neural representation of sound. Although the FFR f0 is widely thought to arise from a combination of subcortical sources, a contribution from the auditory cortex has recently been reported using MEG measures [18]. A stronger FFR f0 amongst fundamentally biased listeners measured at a single electrode could therefore mean a stronger FFR f0 representation in subcortical nuclei and/or in the auditory cortex; methods that allow for source separation will be needed to resolve this question.

This finding also supports our suggestion that inter-subject differences in pitch representation (or precursors to it [49]) in part account for the observed variability in the FFR f0. While there was no unified group effect on f0 strength of the presence of energy at the f0, many individuals' FFR f0 strength did react to the difference in physical properties of the stimulus, albeit not in a consistent direction across the sample, indicating that the difference in timbre between MF and FP tones is likely represented within the FFR.

### Electrode montage affects FFR f0 strength

By comparing electrode montages in common use for FFR recordings (Cz-averaged mastoids and Fz-C7), we explored a less cognitive yet equally relevant source of variability: geometry. It is quite possible that both inter-individual differences and the bi-directional differences observed between conditions and across subjects in many studies may in part be explained by signal projected from multiple cortical and subcortical sources combining with different relative strengths at the electrode site according to differences in brain morphology, head shape, and skull thickness. Analysis of a third (horizontal) electrode montage, which has been used as evidence for a brainstem origin of the FFR f0 to MF stimuli, suggests that earlier findings [53] may have resulted from auditory nerve activity, which may contribute to the recorded FFR signal in certain experimental paradigms and to different degrees using various montages [60]. Future work on inter-individual differences, particularly in explaining the bi-directional differences observed in many studies, might be well-served by methods such as MEG that allow for the contributing sources to be spatially separated [18].

### Voluntary switching between perceptual modes has FFR-f0 and gamma-band neural correlates

In Experiment 2, we showed that if subjects are provided with missing fundamental stimuli that have been customized to take into account their pre-existing biases, their perception of pitch corresponding either to the MF or to the spectrum can be brought under conscious control. Reversible mode perception was previously not considered to be possible, as only a unidirectional spectral-to-fundamental training effect had been observed [38,39]. We then showed that the top-down process of selecting between perceptual modes influences both the strength of the FFR f0 and the strength of low gamma band activity, which are also novel findings (although gamma band changes have been observed during visual perception of reversible or ambiguous figures [72]).

A larger PLV f0 in the fundamental listening condition was related to greater accuracy on a behavioural task that was designed to measure ability to hear in both perceptual modes. Although the mechanism by which perception-related f0 strength change takes place is not yet clear, these findings are consistent with the idea that a stronger f0 represents a relative enhancement in the auditory system [8,59], or at least in its ability to separate and manipulate potentially behaviourally relevant aspects of sound. When considering the finding that the FFR f0 may be affected by top-down influences in conjunction with the considerable evidence for a cortically-based pitch extraction mechanism [30], it seems likely that it is the cortical component of the FFR f0 [18] that is being modulated; whether online modulation and long-term training act on different FFR f0 generators remains for future work.

### Interpretation

Our findings suggest a possible explanation at the neural level for inter-individual differences in f0 perception bias, as well as the observations that listening context affects subsequent mode perception [31,36]: rather than a permanent unidirectional process of plasticity [38], both information about the spectral energy and the periodicity of the waveform's envelope might simultaneously be preserved in the auditory system, perhaps because they are useful in different auditory tasks. The idea that multiple streams of information are represented in different neural populations within the auditory cortex that can be used selectively to meet task requirements has been proposed within the context of spatial auditory stream segregation [73]; a similar mechanism might be at work here and could be a more general characteristic of auditory system organization. Whether one or the other of fundamental or spectral representations is more easily or spontaneously accessed may be affected by experience.

In these experiments, the role of long-term experience, specifically musical training, is observed in the perceptual bias as in previous work [34], but is not clearly represented in the neural correlates of perceptual bias and perceptual mode switching. We did not find clear relationships between measures of musicianship and f0 strength in either study, which has been found in some studies 8,21,22] but not others [23,24]. One possible explanation is that our samples included a wide and heterogeneous selection of musical experience. Considering that group effects of periodicity encoding are inconsistently observed even in studies with groups selected specifically for extremes of musicianship [8,21–24,74], this may reflect that musicianship is only one of several sources of variability. These data address the relationship between FFR and perceptual variability, but not the causes of differences in the FFR and how they relate to perception. Differences in frequency selectivity of the basilar membrane and in the production of distortion products as a result of physical interaction with variable sound levels may also influence the neural representation of sound, and therefore perception. The relative influences of central processes and the peripheral auditory system on perception remains to be addressed. Nonetheless, while peripheral processes may play a role in system bias, they cannot be a cause of the findings in Experiment 2, wherein distinctions were found in the FFR f0 to identical stimuli which the subject perceived in each mode.

Future studies of how long-term musical training may influence both perceptual bias and its f0 correlates could use professional musicians of matched high levels of experience, such as pianists who tend to be f0 listeners, and woodwind players who tend to be spectral listeners [33]; relationships with non-musical experience such as knowledge of tonal languages could also be evaluated [75]; as well as relationships to specific auditory skills such as absolute and relative pitch. Longitudinal designs could be used to assess causality, both before and after musical or linguistic training, and also to study the apparent increase in perceptual flexibility that takes place within an experimental session. Nonetheless, the current work reflects the probable heterogeneity of musical experiences characteristic of most samples in which we are attempting to explain f0 variability; musical training is likely to be one of many influences of biology, experience, methodology, and top-down activity on f0 strength.

Although the difference between conditions in FFR f0 PLV and gamma was strongly correlated, they showed different patterns with respect to listening condition and in relation to behavioural measures. Unlike the f0, which showed a range of effects of condition in both directions (f0 > SP, SP > f0) across subjects, the gamma band activity showed an increase in the f0 condition, on average, across subjects. The gamma difference did not show the same relationship to the behavioural measure as the f0, either; it did not clearly covary with perceptual switching accuracy. The FFR is thought to reflect the fine temporal and spectral properties of the stimulus and is generated in multiple nuclei in the brainstem [2], as well as in the auditory cortex [18]. Gamma activity is thought to be a building block of electrical activity in the brain that allows functional coupling or synchronization of spatially distributed brain tissue, in the service of multiple functions including sound representation, and may also have a brainstem component [40]. By measuring both f0 and gamma, we are likely observing different aspects of the pitch extraction process: FFR f0 strength could reflect how well the periodicity of the sound is encoded, which is then available to higher level processes that either use it or other spectral information that is not represented in the FFR to compute pitch (by default or according to task demands), at which point the inter-region communication requirements of the computation produce gamma band activity. Efferent projections might act to enhance FFR encoding, when needed to meet task requirements.

An emerging viewpoint in the literature proposes that the FFR is a measure of the integrated response of the entire auditory system to sound, and that our knowledge of the auditory system will benefit from studying it as a whole [1,46,76]. To better understand the activities of component parts and their interactions, we believe it will be necessary to use new methods that allow for source separation [18]. We further suggest that the study of the auditory system will benefit from combining measures that have been traditionally studied and reported separately, for example, the FFR as a measure of the brainstem's activity and gamma oscillations or ERPs as a measure of cortical activity, or alternatively, studying the phase and strength of activity across frequency bands over the course of the brain's response to sound. The perceptual switching paradigm presented here is one possibility for whole-system studies on sound processing.

## Conclusion

We show that inter-individual variability in the FFR's f0 representation is related to differences in how individuals perceive sound. In addition to these relatively stable perceptual biases, our results suggest two other factors that contribute to the puzzling degree of variability in FFR f0 representation: online modulation of pitch encoding mechanisms, and the configuration of recording electrodes, the latter likely because of an interaction between standard electrode positioning and differences in head geometry. We showed that short-term top-down cognitive influences can allow access and selection between sound representations if required by a task, though predisposition and learning may favour one representation over another. Clarifying the 'content' of the FFR is relevant for the interpretation of a growing body of FFR-based research results, and may provide insight into the processes which can be enhanced through training and which are at fault in pathology. We have also demonstrated a paradigm that allows the study of top-down effects of the auditory system to simultaneously observe multiple neural correlates, with a view to a modern whole-system approach. This approach may also help to evaluate and improve models of information processing early in the auditory stream for aspects of sound that are important in speech and music, and to develop targeted treatments for individual with specific pathologies.
